# The relationship between social support and work engagement of rural teachers: a moderated mediation model

**DOI:** 10.3389/fpsyg.2024.1479097

**Published:** 2025-01-07

**Authors:** Shengwen Wu, Qiangqiang Xu, Huidong Tian, Rongrong Li, Xiaopeng Wu

**Affiliations:** ^1^School of Teacher Education, Zhejiang Normal University, Jinhua, China; ^2^School of Teacher Education, Tianshui Normal University, Tianshui, China; ^3^School of Education Science, Xinjiang Normal University, Ürümqi, China; ^4^Faculty of Education, Northeast Normal University, Changchun, China

**Keywords:** rural teachers, social support, mindfulness in teaching, work engagement, psychological safety

## Abstract

**Aim:**

This paper aims to investigate the relationship between social support and rural teachers’ work engagement while exploring the mediating effect of mindfulness in teaching and the moderating effect of psychological safety.

**Methods:**

A sample of 866 rural teachers was recruited, in order to complete the Mindfulness in Teaching Scale, Social Support Rating Scale, Psychological Safety Scale, and Utrecht Work Engagement Scale.

**Results:**

The study findings indicate that: (1) social support positively influenced the work engagement of rural teachers; (2) further, mindfulness in teaching partially mediated the effect of social support on rural teachers’ work engagement; (3) psychological safety moderated the second half of the pathway of “social support → mindfulness in teaching → work engagement” while the positive correlation between mindfulness in teaching and work engagement was stronger among rural teachers with high psychological safety.

**Conclusion:**

Social support documented a strong correlation with work engagement while mindfulness in teaching mediated the pathway between the aforementioned variables. Furthermore, psychological safety moderated the second half of the mediated pathway (the link from mindfulness in teaching to work engagement). Hence, the study outcomes reveal the influential mechanism of social support on the work engagement of rural teachers. This finding suggests that we need to further improve the social support system and its effect mechanism in order to improve the rural teachers’ work engagement. At the same time, it is also very important to create a good psychological working environment to ensure that they maintain a good level of mindfulness in teaching.

## 1 Introduction

The construction of rural teachers is not only related to the high-quality development of rural education, but also related to providing important intellectual support for the development of rural society, which is of great significance to the smooth realization of the effective convergence strategy goal of consolidating the achievements of poverty alleviation and rural revitalization. In recent years, under the promotion of a series of favorable policies of the state to strengthen the construction of rural teachers, especially since the implementation of the Rural Teachers Support Plan (2015–2020), and The State Council on Comprehensively Deepening the Reform of the Construction of Teachers in the New Era, and the Action to Revitalize Teacher Education (2018–2022), the status and quality of professional life of rural teachers in China have received much attention from all walks of life. Because education plays a vital role in blocking the intergenerational transmission of poverty and developing poverty-stricken regions, in order to not only become affluent but also get rid of poverty. Work engagement as a kind of working state, can effectively reflect the subjective consciousness of teachers in educational practice (Lan and Liang, 2019). The subjective consciousness of rural teachers is reflected in their profound support, recognition, and willingness to actively seek excellence and innovation, participate in rural education, make continuous efforts to realize the eventual goal of education, and find out the true meaning of rural education (Dai, 2021). These are the key points for rural teachers to improve their work quality and realize rural revitalization, so it is necessary to pay more attention to the state of rural teachers’ work involvement.

Work engagement constitutes the integration of positive emotions and motivation in terms of work, essentially including vitality and dedication (Schaufeli et al., 2006). Teachers’ work engagement refers to the positive attitude and affection depicted by teachers in educational work, which is not only related to the life quality and professional development of teachers but also exerts a significant impact on the education quality and sound development of the students (Perera et al., 2018a). Research shows that teachers’ job involvement not only affects their quality of life, self-efficacy and job satisfaction, but also affects the teaching effectiveness of schools and the physical and mental development of students (Perera et al., 2018b). Nonetheless, teaching represents a highly professional, emotional, and technical profession, therefore, teachers may encounter a wide range of risk factors such as excessive workload, negative interaction with students and colleagues, and onerous demands from parents and the education system (Prilleltensky et al., 2016). The proposed situation is more obvious in the rural education environment with weak teachers, backward educational conditions, and limited professional development (Gao and Li, 2021). Consequently, rural teachers find it difficult to develop a sound sense of professional identity and realization; afterward, their enthusiasm and investment in rural education are not high (Li, 2016). As a result, there is an urgent and substantial need to ascertain how to escape this dilemma and give full play to the positive role of rural education in rural revitalization while consolidating the realizations of poverty alleviation.

### 1.1 The association between social support and work involvement

The job demands-resources model (JD-R) reveals two main factors that influence work: on the one hand, work requirements warrant individuals to invest mental effort and physical strength, and incur even physical and psychological losses; while, on the other hand, resources reduce the work requirements and their consumption, and assist people to attain their work goals, in order to promote their learning and growth (Demerouti et al., 2001), among which prominent resources include work and personal resources. Additionally, social support as an imperative work resource, plays a positive role in individual work performance such as intention to remain (Fu et al., 2022) and subjective career success (Kundi et al., 2022). Aligned with this, social support refers to the supportive resources that an individual yields from other people or society, in order to cope with stress including material and spiritual support to the person (Cohen and Wills, 1985). Besides, social support not only serves as a “buffer” of a person’s negative psychology to deal with psychological crises but also supplements the development of mental health under common circumstances (Fang et al., 2021; Li et al., 2020). Parallel to this, work engagement represents a mental health state displayed by individuals at work, which is also positively impacted by social support (Jolly et al., 2021; Simbula et al., 2023). Notably, social exchange theory holds that there exists a “balanced exchange” between individuals and organizations. Consistent with this, the work behavior of employees shall be adjusted on the basis of support perceived by them; thus, the higher the social support, the higher the job engagement (Cho et al., 2009), as social support from superiors, relatives, and colleagues can help people rapidly adjust their mental and physical state; subsequently, putting them into work (Zeng et al., 2018). For instance, Tuazon inferred that the organizational support offered by schools can make teachers highly involved in their work while cementing their educational beliefs and professional commitments, with a high level of work engagement (Tuazon, 2016). Therefore, this paper proposed hypothesis:

*H1*: Social support positively anticipates the work engagement of rural teachers.

### 1.2 The potential mediating role of mindfulness teaching

From the perspective of the JD-R model, the mediating role of individual resources should also be taken into account when investigating the possible linkage between social support and teachers’ work engagement (Wolter et al., 2019; Zeng et al., 2018). Mindfulness serves as a distinct and critical internal resource (Fisher et al., 2019); consistently, the positive influence of mindfulness on stress relief and emotional improvement is receiving increasing attention (Googhari et al., 2022; Zhang et al., 2021; Zhang et al., 2019). Further, mindfulness also exhibits a certain impact on the work behavior or state of an individual (Li, 2022; Liu et al., 2020). Reportedly, prior studies establish that mindfulness plays a mediating role in the relationship between social support and job burnout (Xie et al., 2022). Since job burnout and work engagement represent two opposite work states, therefore mindfulness may also play a mediating role in the linkage between social support and work engagement. This phenomenon can be explained from the viewpoint of conservation of resources theory (COR). Particularly, COR theory highlights that the acquisition of initial resources shall bring more resources to individuals while the scarcity of resources shall lead to further resource depletion in the future (Hobfoll, 2001). Meanwhile, sustained attention as well as attention to the non-judgmental acceptance contained in mindfulness also necessitates the investment of cognitive resources or the supplement of other resources (Zhang et al., 2017). Given the intake of social support as an external resource, individuals can experience more positive emotions and possess more time and energy to pay due attention to their existing self-state. In other words, social support can address the basic psychological needs of people while making their consciousness focus and attention on the present matters (Liu and Xin, 2019; Shin and Gyeong, 2023). On the same note, empirical studies also illuminate that social support improves the mindfulness level of people (Reb et al., 2015; Wilson et al., 2022). In contrast, mindfulness permits individuals to focus on their internal experiences and external events in a non-judgmental manner (Dane, 2011); thereby, allowing them to not only eradicate the interference of adverse emotions but also optimistically participate in their work. In addition, individuals with high mindfulness have access to a higher amount of internal psychological resources such as high attention, work focus, and objective evaluation which help improve individual job engagement (Liu et al., 2020). Empirical studies also establish that employee mindfulness exerts a positive effect on work engagement.

Although, most of the aforementioned studies focus on the internal mindfulness of a person while research analysis on interpersonal mindfulness which presents the ability to detect the behavior and consciousness of others, is relatively lacking; however, mindfulness in teaching can supplement the proposed deficiency (Ma et al., 2022). As a distinct mindfulness mode of personal and interpersonal behavior exercised by teachers in teaching practices, mindfulness in teaching includes intrapersonal and interpersonal mindfulness. Correspondingly, the former reflects the teachers’ ability to analyze the present and non-judgmental awareness of their own feelings, thoughts, and behaviors in teaching while the latter reflects the teachers’ capacity to remain open, accept situations, and not instantly react to students in teacher-student interaction (Frank et al., 2016). Obviously, mindfulness in teaching practices not only enables teachers to possess an explicit comprehension of their own and students’ state in teaching but also become aware of and accept the needs and emotions of both sides of teacher-student interaction (Ding et al., 2020), which is conducive to teachers, in order to make timely and appropriate self-adjustment in teaching activities; thereby, maintaining a sound emotional state and improving the quality of teacher-student interaction (Wang and Pan, 2023). In line with the current literature and aforementioned analysis, mindfulness teaching positively impacts the work engagement of rural teachers (Wang and Tian, 2024; Moyano et al., 2023).

It is worth noting that the current research only focused on the relationship between social support, mindfulness teaching, and work engagement, and it is not clear whether there is any link between the three. Especially in the process of tracing the issue of “how to motivate rural teachers’ work engagement” in recent years, the research mainly focused on the influence of factors such as teachers’ characteristics, work characteristics (Dehaloo and Schulze, 2013) and organizational environment on rural teachers’ work engagement (Tuazon, 2016), and few studies have explored the influence of teaching mindfulness on rural teachers’ work engagement from a metacognitive perspective. Teaching mindfulness is a key psychological resource to maintain teachers’ occupational health and has positive effects on their work vitality and concentration (Frank et al., 2016). Whether the stimulation of psychological resources and the play of their effectiveness require the intake of external capital, such as social support, remains unclear. However, due to the lag of education results and the complexity of professional requirements, rural teachers need psychological care and external support resources. Therefore, based on the above findings, this paper proposed hypothesis:

*H2*: Mindfulness in teaching serves as the mediating variable between social support and the work engagement of rural teachers.

### 1.3 The potential moderating role of psychological safety

In addition, based on the field theory, employees in organizations are embedded in the environment and their own characteristics impact their attitudes and behaviors (such as personality and motivation) and situational factors (such as interpersonal relationships and organizational environment; Wang and Tian, 2024). Certainly, psychological safety refers to the employee’s perception of the consequences of interpersonal risks in the work condition. In specific, the employees without restrictions articulate themselves in the work without concern regarding the adverse influences on them (Edmondson, 2002). As a result, employees can effectively embody their state of interpersonal relationships and organizational environment. Thus, it is of vital significance to explore whether psychological safety affects the individual trait of mindfulness in teaching when it exerts a significant effect on the work engagement of rural teachers. On the one hand, the psychological safety realized by individuals in their work ascertains their willingness to invest cognitive, emotional, and physical energy in their work (Basit, 2017). Meanwhile, psychological safety discloses the stimulating effect of individual safety needs on work motivation and employees’ performance (Zou and Yin, 2017). On the other hand, psychological safety reflects the cognitive needs of a person for self-ability and environment. Both mindfulness and psychological safety stress the individual’s awareness and control of the interaction between oneself and the external environment. Such a sense of control not only uplifts the positive influence of success but also lowers the negative effects of failure (Bandura et al., 1999). Alternatively, a high level of mindfulness in teaching makes teachers more engaged in their work. Under the impact of high psychological safety, this positive influence may be more obvious. In contrast, it is convenient to create workplace rejection or colleague trust crisis when individual psychological safety is low, therefore, significant psychological resources should be consumed in evaluating and adjusting psychological safety (Lee et al., 2016). Consequently, there is a dire need to ascertain whether the positive impact of mindfulness in teaching on work engagement be sustained when teachers transmit to teacher-teacher interaction after class from teacher-student interaction in the classroom. The inverse predictive influence of mindfulness teaching on negative work mood is only profound in rural teachers with high psychological security which is consistent with the narrative of human-situation interaction theory (Tian et al., 2024). Noticeably, psychological safety creates an organizational environment of mutual trust and respect for individuals while the increment of psychological resources produced by it makes individuals more active in terms of their work (Spreitzer et al., 2012). Meanwhile, the positive emotions of individuals with high psychological safety generally overcome the negative emotions, consequently, exhibiting confidence and relaxation (Senejani et al., 2016), which also makes it convenient for rural teachers to enter the mindfulness state of teaching, improving the level of work engagement. Consistent with this, this study proposed hypothesis:

*H3*: Psychological safety moderates the second half of the path in the model of social support impacting the work engagement of rural teachers through mindfulness in teaching.

**Figure 1:**
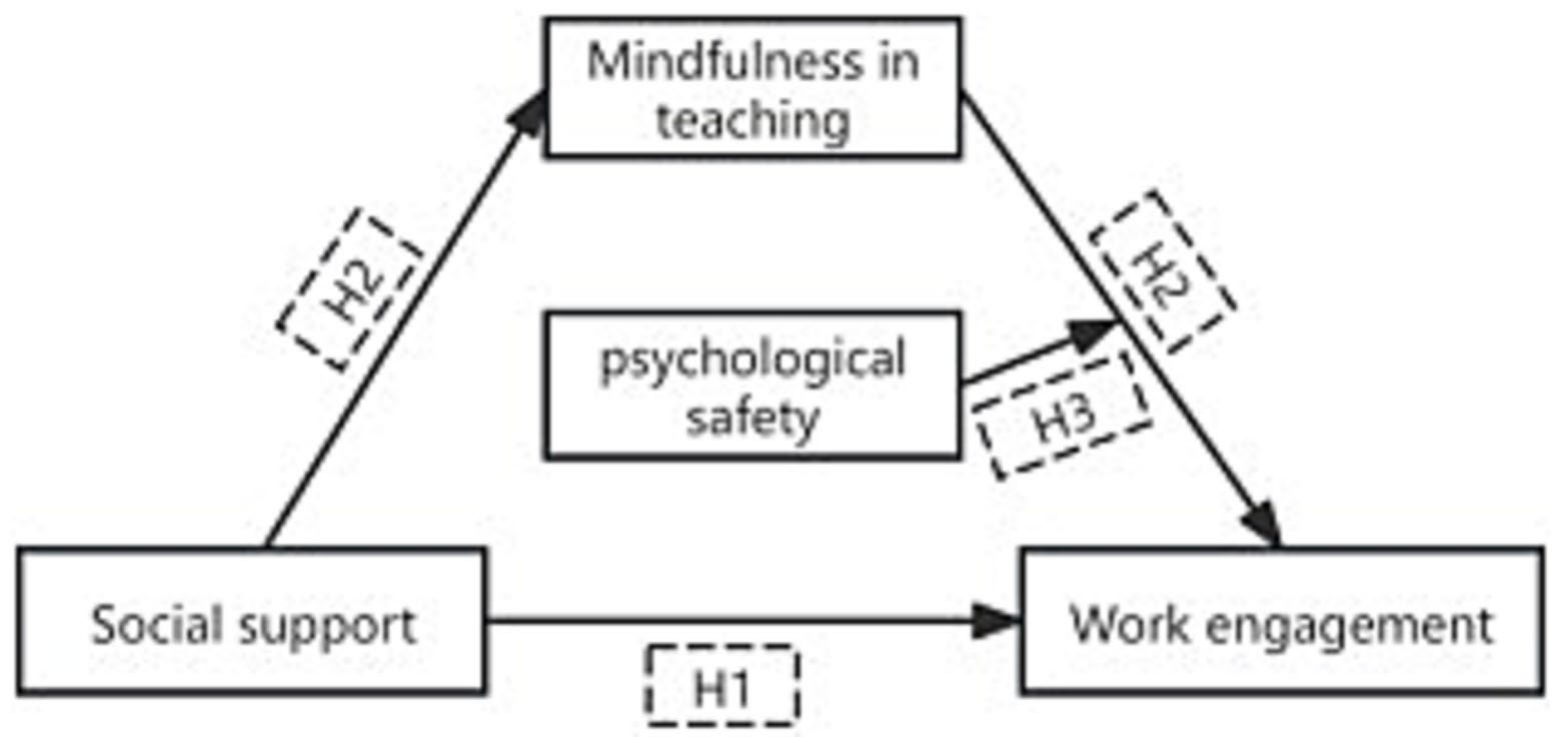
The proposed hypothesis model.

Based on this, a mediated model is built (Figure 1 illustrates the hypothetical model). Based on the potential effects of gender, teaching age, and teaching period on teachers’ work engagement (Li, 2019), the researchers treat the aforementioned variables as control variables.

## 2 Methods

### 2.1 Participants

The researcher or the investigator’s client went to the village school of the respondent and distributed questionnaires to the teachers on the spot after contacting the school of the respondent and obtaining formal permission. All the participants were instructed to anonymously fill out the questionnaire forms in an independent and truthful manner. Using convenient sampling method, 940 rural teachers from Lanzhou, Tianshui, Longnan, Zhangye, Pingliang, Chenzhou, Changji were distributed questionnaires. These prefecture-level cities and states involve different regions of southern and northern in China, suggesting a broad demographic representation. Invalid questionnaires in the recovered questionnaires were removed based on (1) respondents presenting obvious regularity in answering, such as the cycle of “1, 2, 3, 4, 5”; (2) respondents choose extreme options for all questions, such as the highest or lowest score of all questions; (3) there are two or more items missing filling. Finally, with an effective 866 subjects as well as an effective rate of 92.13%. Out of the total 866 valid subjects, 435 were male and 431 were female subjects; likewise, there were 355 primary-school teachers, 364 junior high school teachers, and 147 senior high school teachers. In addition to this, there were 166 individuals with 1–3 years of teaching experience, 206 people with 4–10 years of teaching experience, 277 teachers with 11–20 years of teaching experience, and 217 persons with more than 20 years of teaching experience.

### 2.2 Measures

#### 2.2.1 Social support

Revised by Xiao (1994), the social support rating scale was employed in this study. This scale comprises 10 items which are classified into three key dimensions, namely: objective support, subjective support, and support utilization. Particularly, the higher the score, the higher the social support. Further, the Cronbach’s *α* coefficient of this scale stood at 0.66.

#### 2.2.2 Mindfulness in teaching

Mindfulness in teaching was gaged by the Chinese version of the Mindfulness in Teaching Scale proposed by Frank et al. (2016) and revised by Ding et al. (2020). Explicitly, this scale contains 16 items in total, which are categorized into 2 main dimensions including interpersonal mindfulness and intrapersonal mindfulness. On the basis of a 5-point scale, some of the reverse scale questions were converted by the researchers. Thus, the higher the score, the higher the level of mindfulness in teaching. Finally, the Cronbach’s α coefficient of this scale was reported to be 0.77.

#### 2.2.3 Psychological safety

Psychological safety was estimated by the Chinese version of the Psychological Safety Scale compiled by May et al. (2004) and revised by Li and Yan (2007). This scale contains 5 items in total and employs the 5-point scoring method. Once the reverse scoring is converted, the higher the score, the stronger the psychological safety. Lastly, the Cronbach’s α coefficient of this scale was 0.72.

#### 2.2.4 Work engagement

In this study, a simplified version of the Work Engagement Scale prepared by Schaufeli et al. (2006) was incorporated to evaluate work engagement. The proposed scale includes 9 items in total, including 3 major dimensions, namely: vitality, dedication, and concentration. Using a 7-point scale (0–6 from “never” to “always”), higher scores infer higher levels of engagement. Meanwhile, the Cronbach’s α coefficient of this scale was reportedly 0.92.

### 2.3 Data analysis

SPSS 21.0 and PROCESS 3.5 were employed to analyze the gathered data, in order to evaluate the proposed hypothesis and explore the possible correlation between social support, mindfulness in teaching, psychological safety, and work engagement of rural teachers. Among them, SPSS was utilized to conduct questionnaire reliability and validity tests, correlation analysis, and common method deviation tests. Additionally, PROCESS was adopted for the moderated mediation model test.

## 3 Results

### 3.1 Common method bias

This article adopts a questionnaire survey approach, which is prone to a common method bias (CMB). Therefore, the study questionnaires were inspected for possible common method bias using Harman’s single-factor test before statistically analyzing the data. Accordingly, the study identified 7 factors whose eigenvalues were >1 while the first factor of the amount of variation explained stood at 18.28%, which was less than the critical standard of 40%. This implies that there was no severe common method bias.

### 3.2 Descriptive statistics and correlation analysis among the variables

In this paper, descriptive statistics and correlation analysis were conducted on the research data. Consistently, Table 1 populates the mean, standard deviation (SD), and correlation coefficient of each variable. Independent Sample *t*-Test presented the following implications: on the one hand, the scores of male teachers in mindfulness teaching [*t*_(840)_ = 2.79, *p* = 0.005, Cohen’s d = 0.19] and psychological safety [*t*_(864)_ = 2.88, *p* = 0.004, Cohen’s d = 0.19] were significantly lower, as compared to those of female teachers; while, on the other hand, the scores of social support [*t*_(864)_ = 2.62, *p* = 0.009, Cohen’s d = 0.18] and work engagement [*t*_(859)_ = 2.08, *p* = 0.038, Cohen’s d = 0.14] were reported to be significantly higher than those of female teachers. The results of correlation analysis suggest that social support was significantly positively associated with work engagement (*r* = 0.25, *p* < 0.001) and mindfulness in teaching (*r* = 0.17, *p* < 0.001); further, mindfulness in teaching demonstrated a significant positive relationship with work engagement (*r* = 0.33, *p* < 0.001).

**Table 1:** Descriptive statistics and correlations among variables.

	*M*	*SD*	1	2	3	4
1 SS	4.17	0.87	1			
2 MIT	3.75	0.47	0.17^***^	1		
3 PS	3.53	0.55	0.08^*^	0.68^***^	1	
4 WE	4.28	1.12	0.25^***^	0.33^***^	0.26^***^	1

M, Mean; SD, Standard deviation; SS, Social support; MIT, Mindfulness in teaching; PS, Psychological safety; WE, Work engagement. *p < 0.05, **p < 0.01, and ***p < 0.001.

### 3.3 Moderated mediation effect analysis

All variables were standardized and the moderated mediating effect was studied by SPSS PROCESS. Likewise, the model in Figure 1 was analyzed using a bias-corrected percentile Bootstrap approach based on the 5,000 replicates.

According to Hayes (2017), as well as Wen and Ye (2014), Model 4 of SPSS Macro-program PROCESS was first employed to test the mediating role of mindfulness in teaching between social support and work engagement. Correspondingly, the derived results are depicted in Table 2. Once gender, teaching period, and teaching years are controlled, social support displays a significantly positive effect on mindfulness in teaching (*a* = 0.16, *SE* = 0.03, *p* < 0.001); simultaneously, social support and mindfulness in teaching entered the regression equation. Remarkably, social support establishes a significantly positive impact on work engagement (*c*’ = 0.17, *SE* = 0.05, *p* < 0.001). Additionally, mindfulness in teaching significantly positively explains work engagement (*b* = 0.28, *SE* = 0.03, *p* < 0.001). It is evident that mindfulness in teaching plays a partially mediating role between social support and work engagement. Similarly, the Bias-corrected percentile Bootstrap test also proved that mindfulness in teaching exerted a noticeable mediating effect between social support and work engagement (*ab* = 0.04, BootSE = 0.01, 95% confidence interval [0.02, 0.07]). Finally, the proportion of mediation influence to total effect stood at ab/(ab + c’) = 19.05%.

**Table 2:** Mediation analyses.

Regression equation		Fits the index			Regression coefficient	
Outcome variable	Predictor	*R*	*R* ^2^	*F*	Β	*t*
MIT	SS	0.26	0.07	15.89^***^	0.16	4.74^***^
WE	MIT	0.41	0.17	34.68^***^	0.28	8.73^***^
	SS				0.17	5.44^***^

MIT, Mindfulness in teaching; SS, Social support; WE, Work engagement. **p < 0.01 and ***p < 0.001.

**Figure 2:**
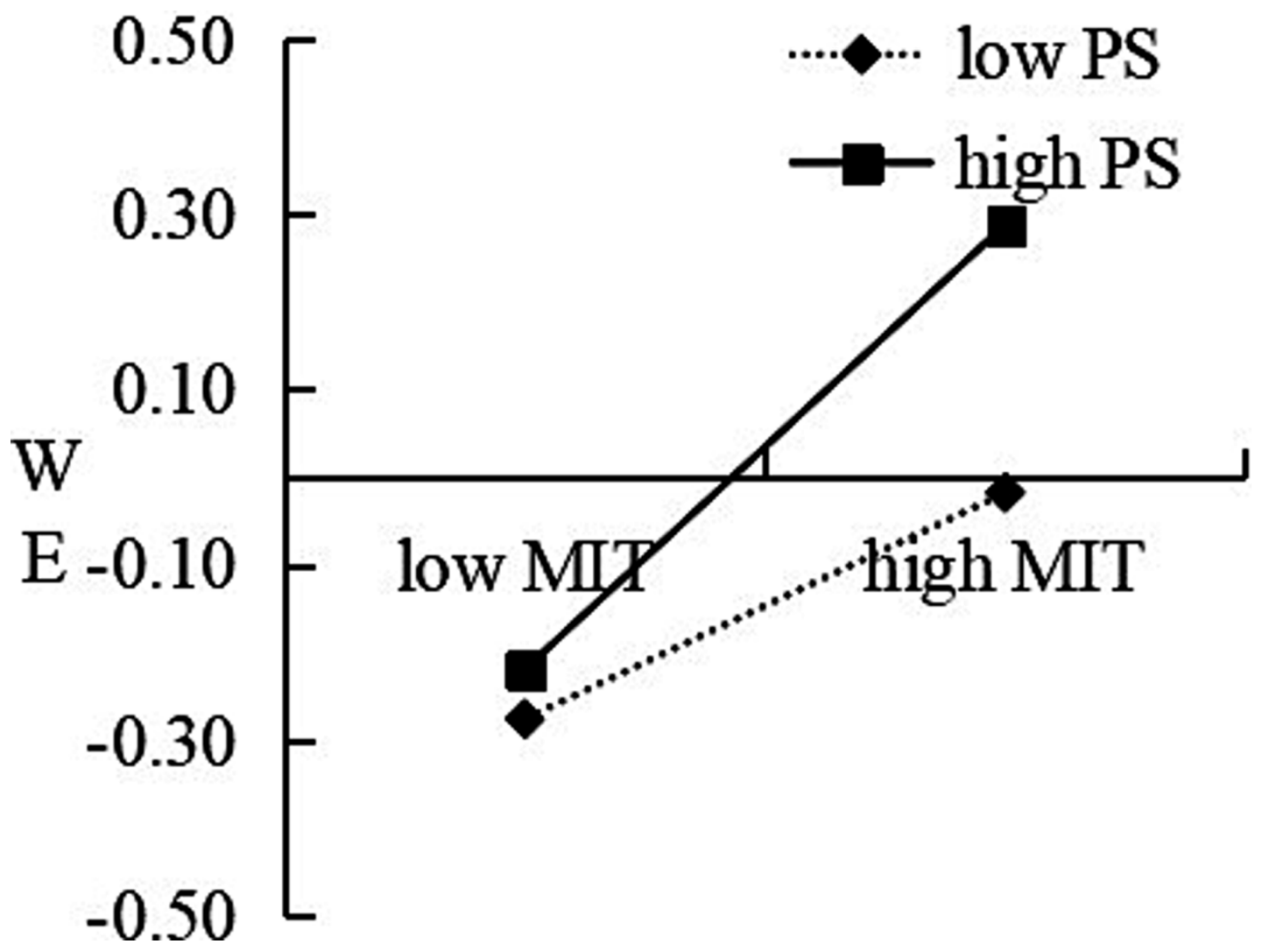
Interaction between psychological safety (PS) and mindfulness in teaching (MIT) on work engagement (WE).

A moderated mediating effect analysis was performed using Model 14 in the PROCESS program (Hayes, 2017). The relevant results suggest that the interaction of mindfulness in teaching and psychological safety significantly anticipated work engagement after controlling gender, teaching period, and teaching years, *β* = 0.07, *p* < 0.001, △*R*^2^ = 0.01, which illuminate that psychological safety could moderate the correlation between mindfulness in teaching and work engagement. In order to more intuitively explore the moderating effect of psychological safety on the correlation between mindfulness in teaching and work engagement, psychological safety was categorized into high psychological- and low psychological safety groups based on the average plus or minus 1 standard deviation (SD), and the interaction diagram was plotted as per the work engagement scores corresponding to + or −1 SD of mindfulness in teaching of diverse psychological safety. As illustrated in Figure 2, mindfulness in teaching exerts a significant positive impact on work engagement for the low psychological safety group (*b*_simple_ = 0.14, *SE* = 0.05, *t* = 2.81, *p* = 0.006, 95% CI [0.04, 0.23]); further, the positive predictive influence was stronger in the high psychological safety group (*b*_simple_ = 0.27, *SE* = 0.05, *t* = 6.01, *p* < 0.01, 95% CI [0.18, 0.36]).

In summary, the moderated mediation model proposed in this study was supported by empirical evidence. Mindfulness in teaching played a mediating role between social support and job engagement, and the mediating role was moderated by psychological security in the second half.

## 4 Discussion

### 4.1 Summary

Referring to the field theory and job demands-resources model, this paper explored the linkage between social support and work engagement among rural teachers in China while examining the mediating role of mindfulness in teaching and the moderation effect of psychological safety. Evidently, social support positively predicted the work engagement of rural teachers which is in line with the current research outcomes (Inggamara et al., 2022; Tuazon, 2016). This implies that social support as a valuable work resource, is of vital significance to the work attitude or behavior of rural teachers. From interpersonal communication, the proposed work resources extend emotional and instrumental social assistance to teachers, in order to ensure that their basic needs can be met. Afterward, teachers shall possess a sense of identity in educational work from the psychological level; thereby, leading to more recognition and investment in educational work; conversely, when work or family gives less support to teachers, they shall feel alienated from their work (Merida-Lopez et al., 2022). At present, rural teachers are still faced with multiple difficulties such as high living pressure, commonly poor salary and treatment, heavy teaching and non-teaching work, and blocked and sensitive interpersonal affiliation (Sun and Lin, 2014; Zhou, 2021). To a certain extent, these specifics eradicate the enthusiasm and contentment of rural teachers. Thus, these teachers urgently warrant support from all walks of life to offer them strong external resources, in order to cope with various pressures, boost or reshape their educational beliefs and confidence, and thereafter invest in rural education.

This article concludes that social support also affected work engagement through mindfulness in teaching. This is similar to the present research (Fathi et al., 2023). Parallel to this, social support positively anticipated an individual’s level of mindfulness (Fabris et al., 2023) while mindfulness exhibited a positive impact on their work engagement since social support can enable individuals to experience more positive emotions, and support them cope with diverse routines or work problems, to a certain extent. This enables the individuals to not only concentrate on themselves but also be aware of the present consequences (Zhang et al., 2023). The aforementioned mindfulness of focus on the present state helps people return to the original state of mind, adjusting their emotions and psychology, recovering from the work pressure, and devoting themselves to work (Liu et al., 2020). It is pertinent to mention that the inclination, significance, and concentration of work implied by teacher’s work engagement signify the overall state perception and engagement of teachers to their work (Perera et al., 2018a), whereas mindfulness in teaching represents the behavior performance or work characteristics of teachers in certain teaching work, which not only carries the common attribute of mindfulness but also acts as the substantial embodiment of their work state or work behavior. In addition, the mediating effect of mindfulness in teaching confirms that social support reflects an indirect influence on a person’s long-term and overall work behavior or state (work engagement) through the individual’s existing specific work performance or characteristics (mindfulness teaching) which captures the mechanism of social support on work behavior performance of people to a certain extent. From the perspective of conservation of resources theory, social support maintains the positive effect of mindfulness in teaching on rural teacher’s work status by supplementing resources in their work field. Since the awareness, concentration, and regulation embodied in mindfulness in teaching shall consume a person’s cognitive or emotional resources, family support and workplace can significantly supplement related resources for rural teachers in time.

The study results also posit that psychological safety moderated the second half path of the mediating model of social support affecting work engagement of rural teachers through mindfulness teaching. This infers that the positive prediction of mindfulness teaching on work engagement is stronger in teachers with high psychological safety. This is similar to the conclusion drawn in past studies, i.e., the impact of mindfulness teaching on negative work emotions is only noticeable in rural teachers with high psychological safety (Tian et al., 2024). This is also aligned with the standpoint that individual traits need to take into account the contextual factors when explaining individual behavior pointed out by person–situation interaction theory (Woodman et al., 1993). Simultaneously, the organizational atmosphere of mutual trust and respect shaped by psychological safety creates psychological resource increment for individuals and makes them more actively involved in work (Spreitzer et al., 2012). Besides this, people with high psychological safety generally have positive emotions that can overcome negative emotions; thereby, displaying relaxation and confidence (Senejani et al., 2016). Specifically, rural teachers bear significant teaching and non-teaching work due to the conflict between the shortage of teachers and the expansion of non-teaching responsibilities in rural schools (Zhou, 2021). Besides, “migratory” teachers who return to the city after work from rural schools have become the key group of rural teachers in the context of urbanization (Jiang, 2018). Owing to the excessive workload as well as frequent switching between school and family life, rural teachers are spared with no time to take care of their families, therefore, the conflicts between work and social life may become more intense (Cheng, 2021). Meanwhile, psychological safety can be adopted as a resource supplement or resource maintenance, in order to ensure that rural teachers still possess sufficient time and energy to devote to teaching work when dealing with work–family conflicts; thereby, making it convenient for rural teachers with high psychological safety to enter into the mindfulness state of teaching and leading to higher work engagement.

In addition to this, the work field of the teacher predominantly includes the classroom and outside which ascertains that the work engagement of the teacher shall also be affected by the classroom and outside the classroom (Liu et al., 2023). Primarily, mindfulness in teaching represents the characteristic of teachers’ teaching behavior in the classroom, and its enduring effect on their work engagement requires to be secure by factors outside the classroom. Contrary to this, psychological safety permits people to express themselves more authentically at work; thus, feeling more contentment and self-esteem (Newman et al., 2017). As an individual’s positive perception of the external environment, psychological safety becomes an imperative “protective” factor to ensure that the positive impacts of mindfulness in teaching can extend from the inside to outside the classroom; thus, supporting the work engagement of teachers. The proposed moderating effect is also aligned with the “facilitation hypothesis” of the “Pretective-Protective Model,” i.e., one resource (psychological safety) can accelerate the positive impact of another resource (mindfulness in teaching), also termed as the “icing on the cake” model (Ye et al., 2018).

It is worth noting that, although the survey objects of this study involve the southern and northern regions of China, which are representative to a certain extent, due to the different economic development conditions in different regions, the social support rural teachers receive and the characteristics of their own resources are still different. This difference may affect how rural teachers use and rely on their social support, as well as how they dig and pay attention to their own resources. Therefore, it is unclear whether the mechanisms identified in this study are influenced by different regions of development. At the same time, although China’s huge population base makes the work participation and production mechanism of rural teachers in China representative to a certain extent, there are also studies comparing the relationship between social support and work involvement between China and the West, and it is found that the research conclusions in China are basically consistent with the existing research conclusions in the West (Xie et al., 2022). However, we still cannot deny the possible influence of different rural cultures on this conclusion. However, the value of this study also lies in the role model of social support on rural teachers’ work involvement in the context of Chinese culture.

### 4.2 Theoretical and practical implications

From the perspective of the field theory and job demands-resources model, this article explores the mechanism of rural teachers’ work engagement based on the 3 micro-subsystems, namely: work resources (social support), work environment (psychological safety), and individual resources (mindfulness in teaching). This article not only proves that the work involvement of rural teachers is affected by social support but also determines how social support anticipates the work involvement of rural teachers by mediating the role of mindfulness teaching as well as moderating the role of psychological safety. This just confirms the previous theories about mindfulness as a stress coping mechanism for teachers, and teaching mindfulness is a key factor in teachers’ teachingIt is a protective factor that can help them effectively identify and regulate their own consciousness and behavior, get rid of negative emotions and psychological difficulties, and thus show a positive working state. Thus, the study conclusions propose the practical strategy of improving the work involvement of rural teachers and supporting the positive role of rural teachers in rural education and consolidation of poverty alleviation from the viewpoint of social support at the environmental level; on the other hand, the study implications also supports the mindfulness teaching and psychological safety of rural teachers at the personal level.

In the context of the role of social support in promoting and ensuring rural teachers’ subjective consciousness in educational practice, all societal sectors should jointly build the social support guarantee system for rural teachers (Dai, 2021). From the viewpoint of objective support, there is a need to perfect and implement various favorable policies of rural education while ensuring the realization of policy guarantee function. In terms of subjective support, families, society, and educational institutes should enhance the recognition of rural teachers’ social status and allocate them deep support at the level of subjective consciousness. Meanwhile, from the standpoint of support utilization, rural teachers must be promoted to make rational and efficient use of support resources from all sides through system guarantee and subject consciousness.

Secondly, mindfulness in teaching can assist teachers in timely comprehending the dynamic pattern of teaching practices, and trigger the teachers’ initiative to summarize and reflect on the teaching process; thus, creating practical knowledge and “internal experience,” which shall both inevitably and promptly improve teaching quality and professional skills of teachers (Li, 2021). From the perspective of mindfulness teaching’s value in shaping practical and reflective teachers, rural teachers must actively pay due attention to mindfulness teaching, explore the practice model and implication of mindfulness teaching in the domain of education based on elucidating the reasoning of mindfulness teaching, and thereafter nurture practical wisdom and improve their own teaching level, with the aim of avoiding the subjectivity loss and habitual restraint in the professional development of rural teachers. Since practice can help teachers reduce emotional distress by fostering emotional balance and improving attention in the working environment (Li, 2021), it is essential to extend necessary training on mindfulness teaching, both before and after the service of rural teachers, in order to support the connotative development of their teaching profession as well as develop an optimistic, sound, and positive attitude toward lifetime.

Finally, rural teachers may find it difficult to develop a higher psychological safety due to the impact of occupation, community, and interpersonal environment (Tian et al., 2024). As a result, there is a substantial need to not only provide supporting hardware facilities but also pay due attention to humanistic care, and develop a positive and rational psychological environment in the implementation of rural education support plan, in order to enhance the endogenous motivation of rural teachers to invest in rural education system.

## 5 Study limitations

Some shortcomings are related to this study. Firstly, this paper only explores the relationship model between overall social support and work engagement of rural teachers (Talebzadeh and Karatepe, 2020). Nevertheless, the influential mechanism of social support from various sources on individual work behavior is different (Kim et al., 2018). Thus, the follow-up studies can further investigate the mechanism of support from varying sources such as family, friends, colleagues, and organizations on the work performance of rural teachers, in order to analyze their work behavior in the context of the interpersonal ecology of rural teachers. Secondly, urban teachers are different from rural teachers in terms of social support and individual resources, so their use of social support and individual resources may also be different, which may also affect the mechanism of social support’s influence on their work involvement. Therefore, follow-up studies can further explore whether there are differences between urban teachers and rural teachers in the impact of social support on job engagement. The third, there are two main types of mindfulness: state mindfulness and trait mindfulness. The latter reflects the individual’s attention in routine life and the perception of the present state while the former emphasizes the individual experience under mindfulness training (Zhang et al., 2017). In this article, mindfulness in teaching evaluation is the mindfulness trait of rural teachers in teaching. Further studies can further scrutinize the role model of state mindfulness between social support and work engagement. And based on the mode of action of mindfulness teaching, the intervention training course of mindfulness teaching can be added to teacher training, and the intervention effect can be further discussed. In addition to this, the cross-sectional study design of this study cannot fully determine the causal relationship between variables. As a result, future studies can further confirm the study results by using an empirical sampling method or experimental study design. Lastly, the data mainly come from social workers’ subjective reports, which may include some subjects’ memory bias. Future studies may consider collecting data from multiple sources, such as family, friends, leaders, and colleagues, to compare subjective and objective data.

## 6 Conclusion

This paper studied the mediating role of mindfulness in teaching and the moderation effect of psychological safety in the relationship between social support and work engagement of rural teachers. The study results indicate that social support documented a strong correlation with work engagement while the mediating role of mindfulness in teaching mediated the pathway between the aforementioned variables. Furthermore, psychological safety moderated the second half of the mediated pathway (the link from mindfulness in teaching to work engagement). Specifically, the promoting effect of psychological safety was more pronounced at the high level of psychological safety.

## Data Availability

The original contributions presented in the study are included in the article/supplementary material, further inquiries can be directed to the corresponding author.
